# Equity in HTA: what doesn’t get measured, gets marginalised

**DOI:** 10.1186/s13584-017-0162-3

**Published:** 2017-07-10

**Authors:** Richard Cookson, Andrew J. Mirelman

**Affiliations:** Centre for Health Economics, University of York, York, YO10 5DD UK

## Abstract

When making recommendations about the public funding of new health technologies, policy makers typically pay close attention to quantitative evidence about the comparative effectiveness, cost effectiveness and budget impact of those technologies – what we might call “efficiency” criteria. Less attention is paid, however, to quantitative evidence about who gains and who loses from these public expenditure decisions, and whether those who gain are better or worse off than the rest of the population in terms of their health – what we might call “equity” criteria. Two studies recently published in this journal by Shmueli and colleagues suggest that this efficiency-oriented imbalance in the use of quantitative evidence may have unfortunate consequences – as the old adage goes: “what gets measured, gets done”. The first study, by Shmueli, Golan, Paolucci and Mentzakis, found that health policy makers in Israel think equity considerations are just as important as efficiency considerations – at least when it comes to making hypothetical technology funding decisions in a survey. By contrast, the second study – by Shmueli alone – found that efficiency rules the roost when it comes to making real decisions about health technology funding in Israel. Both studies have limitations and potential biases, and more research is needed using qualitative methods and more nuanced survey designs to determine precisely which kinds of equity consideration decision makers think are most important and why these considerations do not appear to be given much weight in decision making. However, the basic overall finding from the two studies seems plausible and important. It suggests that health technology funding bodies need to pay closer attention to equity considerations, and to start making equity a quantitative endpoint of health technology assessment using the methods of equity-informative economic evaluation that are now available.

Across the world, public decision makers responsible for the funding of new health technologies pay close attention to quantitative evidence on the comparative effectiveness, cost effectiveness and budget impact of those technologies [1]. Loosely speaking, we can think of this as evidence about “efficiency”, or getting the biggest sum total health benefit out of scarce resources. However, researchers and policymakers are increasingly interested in finding ways of producing quantitative evidence about broader outcomes relating to “equity”, or fairness in the distribution of health and health care [2–4]. Two studies recently published in this journal by Shmueli and colleagues [5, 6] show why the quantification of equity outcomes is so important, by confirming the old adage: “What gets measured, gets done.” Cost-effectiveness is measured in health technology assessment (HTA), whereas equity is not. It is perhaps not surprising to find, then, that HTA decision-making in Israel is predominantly driven by cost-effectiveness rather than equity [6].

The first study examined the views of senior Israeli health policy makers about the relative importance of three efficiency and four equity criteria [5]. Policy makers were asked to make a series of hypothetical funding choices, in a discrete choice experiment survey designed in a similar way to a previous multi-national study led by one of the authors of this commentary [7]. The study found that Israeli policy makers seem to care at least as much about equity as efficiency – and even more so than policy makers elsewhere in the world. According to a regression analysis of the determinants of their hypothetical choices [5], people directly involved in health technology funding decisions gave roughly equal importance to equity and efficiency criteria (a total estimated weight of 49% for the four equity criteria and 51% for the three efficiency criteria), and other policy makers gave slightly higher weight to equity (56%).

The second study examined the relative importance of these same equity and efficiency criteria as revealed by actual decisions about the public funding of health technologies in Israel [6]. It found that efficiency ruled the roost in practice [6]. The ranking predicted by one efficiency criterion alone – cost-effectiveness in terms of cost per Quality Adjusted Life Year (QALY) – was reasonably well correlated with the actual ranking (correlation coefficient 0.45). By contrast, the ranking predicted by the findings of the first study (i.e. using data on how each technology performs on all four equity criteria and all three efficiency criteria, weighted by their estimated importance) was actually *negatively* correlated with the actual rankings. In other words, if we take these findings at face value, decision makers seemed in practice more likely to choose *less* equitable technologies!

This glaring discrepancy is particularly interesting because Shmueli and colleagues were able to elicit views from 11 former members of the “Basket Committee”, which makes health technology reimbursement decisions in Israel, including four former chairs of this committee. Their sample also included 54 other health policy makers including past and present senior managers from the Ministry of Health, Ministry of Finance, sickness funds, Israeli Medical Association, and hospital directors.

What should we make of these findings? The first question to ask is whether the findings are credible. There are certainly many potential sources of bias and error one could point to in both studies. Quick responses to hypothetical questions in an on-line survey may elicit unreflective “socially desirable” responses – the easy response being that everything matters and all considerations should be equally well considered. By contrast, real decisions may concentrate minds on what matters most under conditions of scarce resources. Also, the concepts of efficiency and equity are hotly contestable, and one can raise all sorts of issues about the selection and definition of the seven criteria in the study.

The seven binary criteria shown in the table were used, with the four criteria on the left falling under equity and the three criteria on the right falling under efficiency.

**Table:** 

Equity criteria	Efficiency criteria
1. The technology is intended for patients suffering from a serious disease (life expectancy is less than 2 healthy years).	5. Cost per Quality Adjusted Life Year: Less than the GNP *[Gross National Product]* per capita.
2. The technology is intended to treat a disease common among children.	6. The Number of patients requiring the technology: more than 100,000.
3. The technology is intended to treat a disease common among the elderly.	7. Individual Benefit: addition of more than 5 healthy years.
4. Funding the technology is required so that the poor can use it.	

There is some logical overlap between the second and third criteria (since a technology for children cannot also be one for the elderly), between the fourth and fifth criteria (since the cost of treatment is related to both cost-effectiveness and whether the poor could otherwise afford to pay privately), and between the fifth and seventh criteria (since cost per unit of benefit is a function of benefit). This co-linearity could potentially bias the regression estimates of the relative importance of each criterion. Furthermore, the third and fourth equity criteria are both ambiguous. Age is partly an efficiency issue rather than an equity issue, insofar as older patients may stand to gain fewer years of healthy life, and it is not clear whether treating the elderly is supposed to be more or less equitable than treating the middle aged. It is also not clear whether the fourth equity criterion relates to the effectiveness of the alternative publicly funded standard of care or the cost of treatment or both; nor is it clear what general equity objective is being invoked – the objective of reducing unfair inequality in financial risk protection, of reducing unfair inequality in the utilisation of care, of reducing unfair inequality in health outcomes, or something else.

Another issue is the relatively small sample of 34 technologies used in the second study, which may not be enough to draw robust inferences about which factors drive decision-making. A previous study in England had a much larger sample and used a regression approach to analyse the determinants of decision acceptance [8]. Interestingly, however, that study similarly found that cost-effectiveness was the dominant criteria for decision-making in England.

One might also worry about various kinds of selection bias. While the number of Israeli policy makers who responded to the survey is impressive (65 out of 147 contacted), it is not clear how representative they are of health policy makers more generally in Israel. The decisions selected in study two all relate to the year 2006/7, while the survey was conducted nearly a decade later. We are told that it was a random sample of 18 accepted and 16 rejected technologies in 2006/7, though no details are given of the randomisation process or the total number of decisions in the full population. More importantly, there is potential for bias in the selection of reported point estimates for the seven decision criteria. In our experience of retrospectively analysing decisions by the National Institute for Health and Clinical Excellence (NICE) in England, reporting of information is patchy and ambiguous. Hence many technology appraisal decisions cannot be included in studies of this kind, or require the analyst to make questionable assumptions about which of many different reported estimate to use. So it would be a sign of exemplary transparency on the part of the Israeli authorities – far better than in England – if enough clear and precise published details were provided on every single technology, not only to include in the random sample, but also to provide unambiguous point estimates on each of the seven decision criteria.

Despite these concerns, it is reasonable to conclude that the study does tell us something useful about the relative importance of equity and efficiency considerations in the minds of decision makers. More research is needed, however, using qualitative methods and more nuanced survey designs to determine precisely which kinds of equity consideration decision makers think are most important and why these considerations do not appear to be given much weight in decision making.

The next question to ask is: so what? Does it matter if there is a mismatch between what health policy makers say and do? It could be argued that the mismatch does not matter, and that cost-effectiveness should remain the dominant criterion in health technology funding decisions irrespective of what policy makers say about equity criteria. We would argue that this mismatch does matter. We agree with Shmueli that the decisions made by policy makers should, as far as possible, reflect their stated values and objectives. We therefore agree that equity considerations are not currently given appropriate weight in health technology reimbursement decisions, both in Israel and more generally across the world, and that ways need to be found of giving them greater priority. Finally, we would go further than Shumeli in arguing that one key way to facilitate this is to quantify equity outcomes so that they receive the same attention in the decision-making process as efficiency outcomes. There are several analytical approaches now available that quantify equity and efficiency considerations and assess the potential trade-offs between them [3]. For example, Norway [9] and the Netherlands [10] already use methods for measuring and valuing severity of illness alongside cost-effectiveness, based on the concept of health shortfall or burden of illness, and we have recently developed methods for measuring impacts on inequality in lifetime health and analysing potential trade-offs with cost-effectiveness, known as Distributional Cost-Effectiveness Analysis (DCEA) [11].

In summary: this pair of studies has a striking and important finding about a potential mismatch between what health technology decision makers say and do about equity. Despite methodological quibbles about the study design, the finding does provide evidence suggesting that equity considerations are not given enough attention in practice. If this mismatch is confirmed in future research, this finding could prove influential in helping policymakers re-shape health technology assessment processes to pay closer attention to equity outcomes, not only in Israel but across the world.

## Abbreviations

DCEA: Distributional cost effectiveness analysis
GNP: Gross national product
HTA: Health technology assessment
NICE: National Institute for Health and Clinical Excellence
QALY: Quality adjusted life year

## References

[1] Daniels N, Porteny T, Urritia J (2016). Expanded HTA: enhancing fairness and legitimacy. Int J Health Policy Manag.

[2] Horton R (2013). Offline: the error of our health technology assessment ways. Lancet.

[3] Cookson R (2017). Using cost-effectiveness analysis to address health equity concerns. Value Health.

[4] Cookson R (2016). Equity-informative health technology assessment – a commentary on Ngalesoni, Ruhago, Mori, Robberstad &amp; Norheim. Soc Sci Med.

[5] Shmueli A (2017). Efficiency and equity considerations in the preferences of health policy-makers in Israel. Isr J Health Policy Res.

[6] Shmueli A (2017). Do the equity-efficiency preferences of the Israeli Basket committee match those of Israeli health policy makers?. Isr J Health Policy Res.

[7] Mirelman A (2012). Decision-making criteria among national policymakers in five countries: a discrete choice experiment eliciting relative preferences for equity and efficiency. Value Health.

[8] Dakin H, et al. The influence of cost-effectiveness and other factors on Nice decisions*.* Health Econ. 2015;24(10):1256–71. 10.1002/hec.3086 25251336

[9] Ottersen T (2016). A new proposal for priority setting in Norway: open and fair. Health Policy.

[10] Van de Wetering E (2013). Balancing equity and efficiency in the Dutch basic benefits package using the principle of proportional shortfall. Eur J Health Econ.

[11] Asaria M (2015). Distributional cost-effectiveness analysis of health care programmes--a methodological case study of the UK bowel cancer screening Programme. Health Econ.

